# Greenery or fear: Site use of Himalayan ibex in a predator-dominant landscape

**DOI:** 10.1016/j.isci.2026.117885

**Published:** 2026-10-22

**Authors:** Hussain Ali, Chris Sutherland, Jaffar Ud Din, Muhammad Ali Nawaz

**Affiliations:** 1 Department of Zoology, Quaid I Azam University, Islamabad 45320, Pakistan; 2 Snow Leopard Foundation, House 71 C, E-11/3, Islamabad, Pakistan; 3 The Snow Leopard Trust, Seattle, WA, USA; 4 Center for Research into Ecological and Environmental Modelling, University of St Andrews, St Andrews, Scotland; 5 Environment Science Program, Department of Biological and Environmental Sciences, Qatar University, P. O. Box 2713, Doha, Qatar

**Keywords:** Himalayan ibex, snow leopard, occupancy, camera trapping, Karakoram, mammal, Pakistan

## Abstract

The Himalayan ibex (*Capra sibirica*), a prized trophy and crucial prey species of the snow leopard *(Panthera uncia*), has increased in abundance alongside snow leopard populations due to ongoing conservation efforts. However, the behavioral strategies that enable ibex to persist in the predator-rich mountain ecosystems of Pakistan remain poorly understood. To address this gap, we investigated habitat use and co-occurrence patterns of Himalayan ibex and snow leopards by using occupancy modeling. Ibex occupancy was positively associated with rugged terrain and higher elevations and negatively associated with distance from roads. Snow leopards showed a preference for rugged habitats. The co-occurrence of these species was mediated by roads. Ibex avoided snow leopard abundant areas by shifting toward more remote locations to reduce predation risk, whereas snow leopards adaptively track these prey movements. This study suggests that protecting remote, heterogeneous landscapes is essential for sustaining mountain ecosystems and predator-prey dynamics in northern Pakistan.

## Highlights

• This study provides understanding of Ibex survival strategies in predator-dominated landscape
• Ibex and snow leopards prefer rugged, elevated, and sparsely vegetated habitats
• Distance to roads was the strongest predictor of predator-prey co-occurrence
• Ibex traded food access for safety; conserving shared habitats can sustain both species

## Graphical abstract

**Figure:**
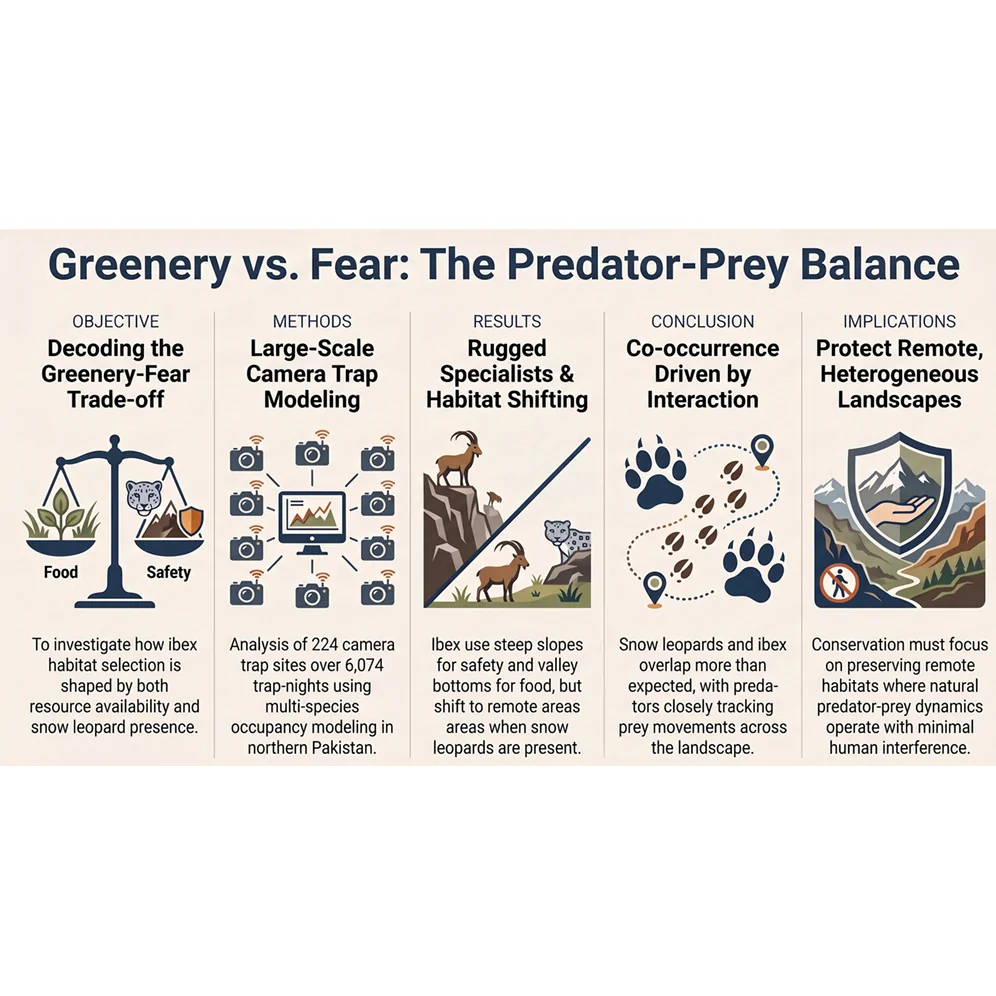

## Teaser

Wildlife behavior; Ecology; Zoology; Animal science

## Introduction

For herbivores, foraging is rarely a simple matter of maximizing energy intake. It unfolds within a landscape where the risk of predation constantly shadows the food search. Predation risk operates through both lethal and non-lethal pathways, shaping animal behavior, spatial distribution, and community structure.^1^^,^^2^ While direct killing is the most obvious outcome, the perceived risk of predation fear can impose even more pervasive costs. Prey modifies their behavior proactively or reactively, increasing vigilance, forming protective groups, or avoiding certain habitats altogether.^3^^,^^4^ These adjustments often force trade-offs between safety and essential activities such as foraging, mating, and rearing young.^5^^,^^6^ Consequently, herbivores do not simply occupy the most productive patches; they continuously balance the need for nutritious forage (“greenery”) against the imperative of avoiding predators (“fear”).

This balance is conceptualized as the landscape of fear, a spatial map of perceived predation risk, where peaks represent high-risk areas with elevated hunting success and valleys serve as safe refuges.^7^ Early versions of this framework treated risk as relatively static, but recent work emphasizes that fear landscapes are dynamic across space and time, shaped by diel, lunar, and seasonal rhythms of predator activity.^8^^,^^9^ Prey may exploit predictable pulses of safety to access resource-rich areas during predator downtimes, effectively flattening the topography of fear.^10^ Understanding how wild ungulates navigate this trade-off is particularly pressing in high-altitude ecosystems, where resources are scarce, environmental harshness is extreme, and predator prey interactions are tightly coupled.

The Himalaya-Karakoram region of northern Pakistan offers a compelling natural laboratory to examine the greenery-fear trade-off. Here, the Himalayan ibex (*Capra sibirica*), the largest and most abundant wild ungulate in the country, serves as both a trophy species for mountain communities and the primary prey of the snow leopard (*Panthera uncia*).^11^^,^^12^ Conservation efforts, including reduced poaching, have allowed ibex populations to recover, with potential knock-on effects on snow leopard numbers. However, the strategies ibex employs to survive in this rugged, predator-rich, yet sparsely vegetated landscape remains poorly understood. Unlike productive savanna or forest systems, the Karakoram’s cold desert supports only patchy, low-biomass vegetation, mainly along river corridors and at lower elevations. In such settings, the classic trade-off may shift: greenery is not abundant but confined to discrete patches, each carrying its own risk profile. For ibex, selecting a site involves weighing not only the quality of forage but also the likelihood of encountering an ambush predator that excels in broken, rocky terrain.^13^^,^^14^

Snow leopards are ambush predators that favor rugged topography, cliffs, and drainage lines to stalk and kill prey.^14^^,^^15^ Their hunting success is tightly linked to terrain complexity and concealment. Ibex, in contrast, rely on their agility, steep escape terrain, and sometimes altitudinal migration to reduce vulnerability.^16^^,^^17^ Yet, both species regularly co-occur, and their spatial overlap is not random but likely mediated by habitat features that either facilitate or hinder predator-prey encounters. A key unresolved question is whether ibex prioritize safety over access to relatively greener foraging areas or whether they risk using productive patches despite higher predator presence. Similarly, it is unclear whether snow leopard space use is driven primarily by its own habitat preferences (e.g., concealment for ambush) or shifts toward areas where prey is concentrated, even if those areas are not intrinsically optimal for hunting.

To address these questions, we used camera-trap data from a large spatial extent in the Hunza and Nagar districts of northern Pakistan, analyzed within a multi-species occupancy modeling framework.^18^^,^^19^ This approach allows us to estimate species-specific habitat associations while accounting for imperfect detection and to test whether co-occurrence patterns deviate from what would be expected based on habitat alone. Specifically, we tested two hypotheses aligned with the greenery-fear dichotomy:

(1) H_1_ (fear-driven ibex selection): reflecting an anti-predator strategy, ibex will select areas that offer escape routes or visibility, in presence of snow leopard, even if such areas have lower vegetation productivity.
(2) H_2_ (prey-driven predator adjustment): in zones of co-occupancy, snow leopards will adjust spatial behavior in response to prey spatial patterns.

By testing these hypotheses, we aim to move beyond a static description of habitat use and instead understand how the interplay of forage availability and predation risk shapes the spatial coexistence of a dominant predator and its primary prey in one of the world’s most extreme mountain ecosystems.

## Results

We deployed 224 camera traps for a total of 6,074 trap-nights. resulting in an average of 27 operational trap-nights Naive occupancy was 0.20 for snow leopards (44 sites; 65 detections) and 0.18 for Himalayan ibex (40 sites; 73 detections), with co-detections at 10 sites. Other wildlife observed included red fox (*Vulpes vulpes*), stone marten (*Martes foina*), weasel (*Mustela* spp.), blue sheep (*Pseudois nayaur*), pika (*Ochotona roylei*), golden marmot (*Marmota caudata*), and snowcock (*Tetraogallus himalayensis*).

Site-specific capture frequencies varied geographically. Khunjerab National Park had the highest detection rates: snow leopards at 20 sites, ibex at 22 sites, and both species co-occurring at 4 sites. In Misgar Valley, snow leopards were detected at 14 sites, ibex at 12, and both species at 4. In Hunza-Nagar, snow leopards were detected at 10 sites, ibex at 6, and both species at 2.

### Site use by ibex

Akaike Information Criteria (AIC)-based model selection of single-species occupancy models retained ruggedness, distance to roads, and elevation as important predictors of ibex site use (Table 1).

**Table 1:** Summary of model selection results for single-species occupancy (step 1) and co-occupancy (step 2) of ibex and snow leopard

Model	AIC	Delta	AIC weight	Cumulative weight	K
**Step 1: ibex**					

1. ψ (Elevation) p (RGN)	507.70	0.00	0.270	0.27	4
2. ψ (Elevation) p (DISRD)	507.82	0.12	0.255	0.52	4
3. ψ (Elevation) p (Elevation)	508.39	0.68	0.255	0.72	4
4. ψ (Elevation) p (.)	509.23	1.52	0.126	0.84	3
5. ψ (Elevation) p (NN)	510.81	3.10	0.057	0.90	4
6. ψ (Elevation) p (NDVI)	511.23	3.52	0.046	1.00	4

**Step 1: snow leopard**					

1. ψ (NDVI) p (RGN)	546.80	0.00	0.228	0.23	4
2. ψ (.) p (RGN)	546.93	0.13	0.213	0.44	3
3. ψ (NN) p (RGN)	547.71	0.92	0.144	0.59	4
4. ψ (DISRD) p (RGN)	547.92	1.12	0.130	0.71	4
5. ψ (DISRV) p (RGN)	548.01	1.22	0.124	0.84	4
6. ψ (Elevation) p (RGN)	548.87	2.08	0.081	0.92	4
7. ψ (RGN) p (RGN)	548.88	2.08	0.080	1.00	4

**Step 2: co-occupancy**					

1. ψ (DISRNE + DISRD + DISRNE) p (Elevation + RGN)	1056.99	0.00	0.333	0.33	10
2. ψ (. +. +.) p (Elevation + RGN)	1057.52	0.53	0.256	0.59	7
3. ψ (RGN + RGN + RGN) p (Elevation + RGN)	1057.94	0.95	0.207	0.80	10
4. ψ (Elevation + Elevation + Elevation) p (Elevation + RGN)	1059.39	2.39	0.101	0.90	10
5. ψ (NN + NN + NN) p (Elevation + RGN)	1061.40	4.41	0.037	0.93	10
6. ψ (DISRV + DISRV + DISRV) p (Elevation + RGN)	1061.59	4.60	0.033	0.97	10
7. ψ (NDVI + NDVI +NDVI) p (Elevation + RGN)	1061.60	4.61	0.033	1.00	10

Covariates: Elevation, elevation; RGN, ruggedness; NN, northness; DISRV, distance to rivers; NDVI, normalized difference vegetation index; DISRD, distance to roads.

Ibex site use was positively associated with ruggedness and negatively associated with both distance to roads and elevation (Table 2). The strong positive relationship with ruggedness (Figure 1A) highlights the importance of steep, rocky terrain as a key habitat feature and likely an important anti-predator strategy. Even at lower elevations, ibex appear to preferentially use steep valley side-slopes rather than relatively flat valley bottoms.

**Table 2:** Parameter estimates of single species occupancy models for ibex and snow leopard in northern Pakistan

	Estimate	Std.error	z value	Pr(>|z|)
**Ibex**				

Ψ (.)	−1.2507	0.629	1.988	0.0468∗
Ψ (RGN)	0.5696	0.3162	1.801	0.0717.
Ψ (DISRD)	−1.1202	0.6382	1.755	0.0792.
Ψ (Elevation)	−1.2822	0.9099	1.409	0.1588
p (.)	−1.0334	0.3723	2.776	0.0055∗∗
p (Elevation)	1.158	0.4612	2.511	0.0120∗

**Snow leopard**				

Ψ (.)	−0.808	0.375	−2.15	0.0313
Ψ (NDVI)	8.401	6.15	1.37	0.172
p (.)	−2.259	0.206	−10.96	6.03E−28∗∗∗
p (RGN)	0.501	0.225	2.23	0.02.58∗

**Co-occupancy models**				

Ψ [ibex] (.)	−4.03	1.692	−2.38	0.0171
Ψ [ibex] DISRD	−3.69	1.825	−2.02	0.043∗
Ψ [SLeopard] (.)	−1.35	0.726	−1.85	0.0639
Ψ [SLeopard] DISRD	−1.12	0.884	−1.27	0.2056
Ψ [ibex:SLeopard] (.)	4.08	2.053	1.99	0.0471∗
Ψ [ibex:SLeopard] DISRD	4.47	2.332	1.92	0.0553
p [ibex] (.)	−1.084	0.336	−3.22	0.0013∗
p [ibex] Elevation	1.04	0.352	2.96	0.0031∗∗
p [SLeopard] (.)	−2.281	0.205	−11.12	0.0000∗∗∗
p [SLeopard] RGN	0.425	0.23	1.85	0.0650

Significance codes: 0, “∗∗∗”; 0.001, “∗∗”; 0.01, “∗”; 0.05, “.”; 0.1, “ ” 1.

**Figure 1:**
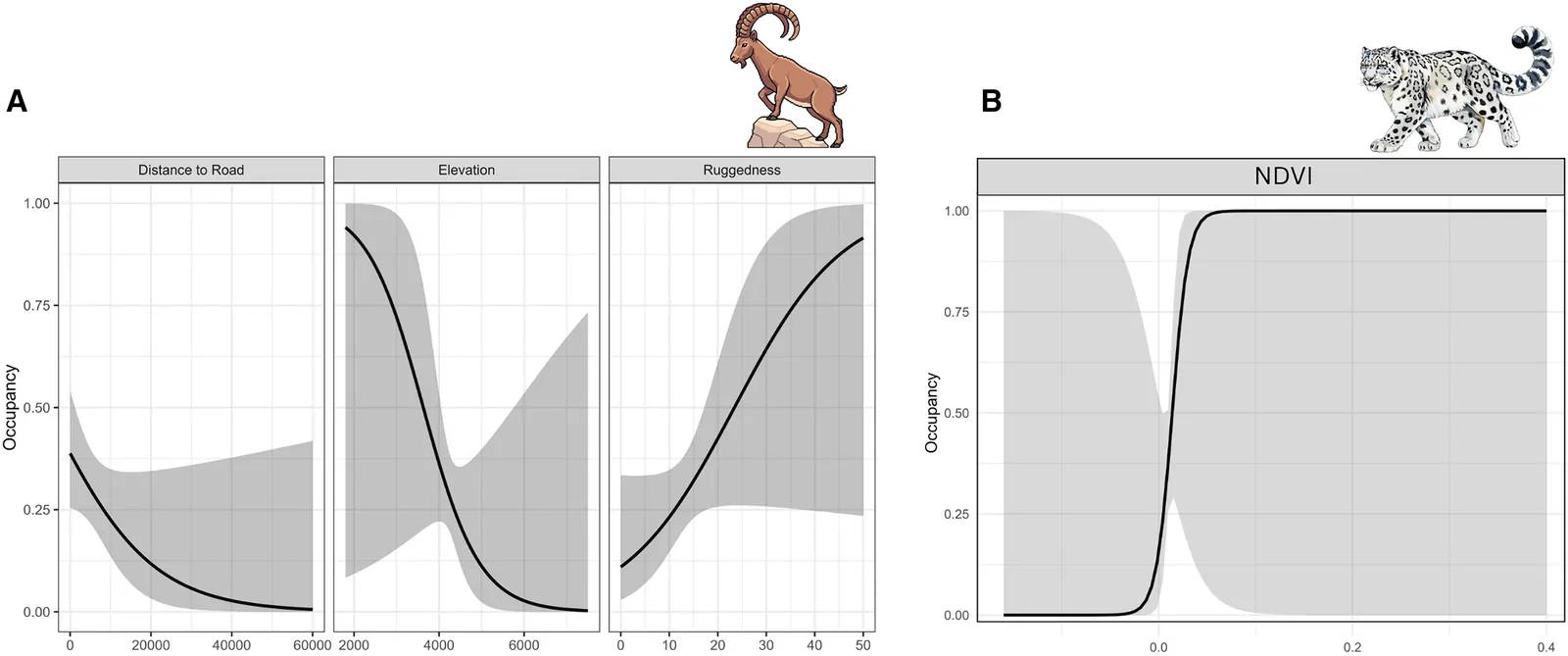
Covariate Effects on Site Use by Himalyan Ibex (A), and Snow Leopard (B) in northern Pakistan

The combined negative effects of distance to roads and elevation (Figure 1A) suggest that ibex are strongly associated with lower-elevation valley systems. Their apparent association with road-accessible corridors should be interpreted cautiously, as roads in mountain landscapes often follow river valleys. Therefore, the observed pattern likely reflects a preference for productive valley habitats where forage and water resources are concentrated, rather than a direct attraction to roads themselves.

Elevation was retained in both the occupancy and detection components of the model, but its effects operated in opposite directions. Occupancy decreased with increasing elevation, whereas detection probability increased. In lower valleys, rugged terrain and denser vegetation may reduce detectability by providing cover and limiting visibility. In contrast, higher elevations are typically characterized by open alpine meadows and scree slopes, where ibex are more easily observed.

Overall, the results indicate that ibex are low- to mid-elevation rugged-terrain specialists. They primarily use steep slopes within valley systems, particularly in areas closer to roads and rivers where resources are likely to be concentrated.

This habitat-use pattern is clearly reflected in the spatial occupancy map (Figure 2A), which shows that 48.0% of the study area falls within the low occupancy class (0.00–0.25), 50.6% within the moderate occupancy class (0.26–0.50), and less than 2% within the high occupancy class (>0.50).

**Figure 2:**
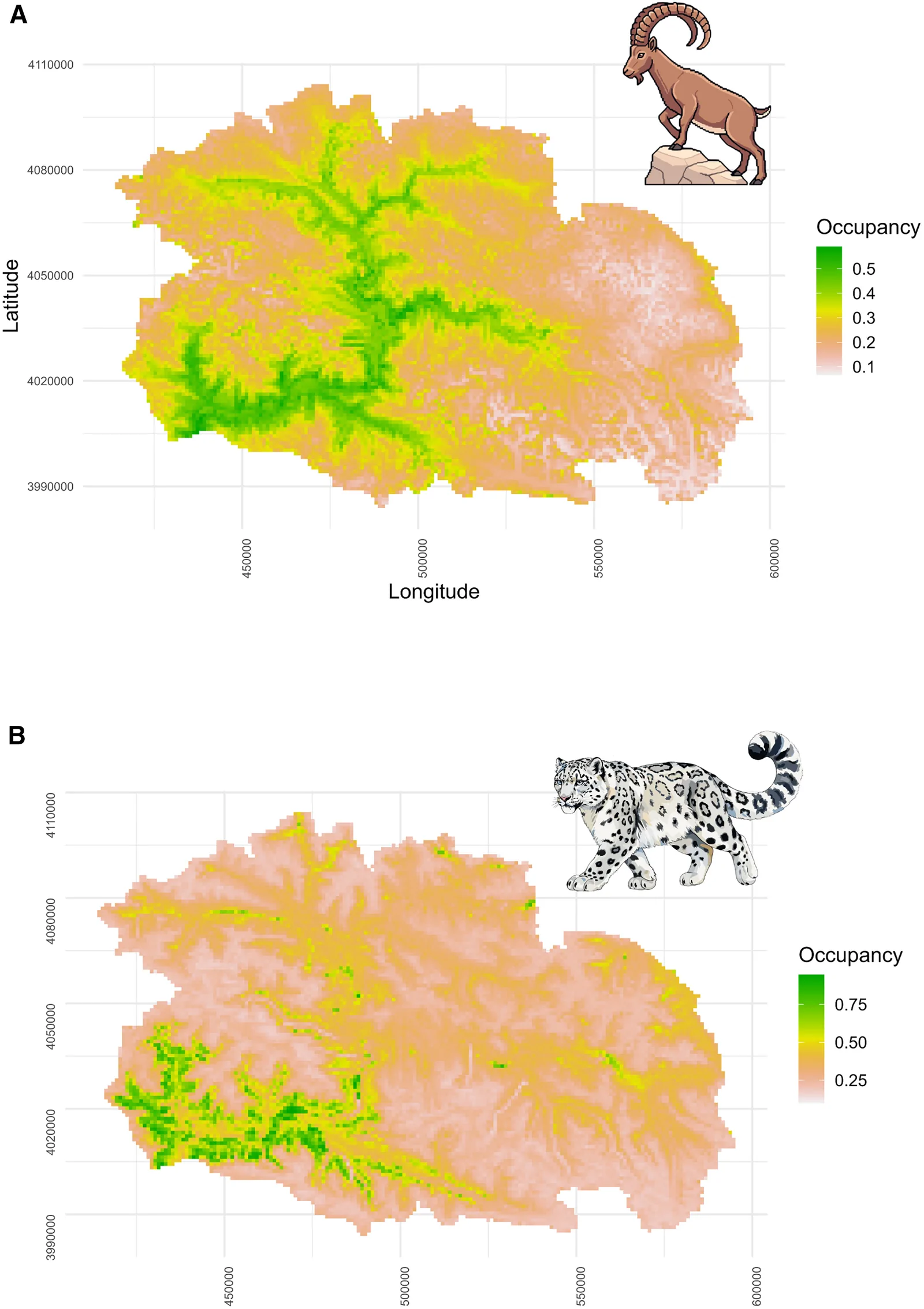
Spatil pattren of Site use by Himalayn Ibex (A) nad Snow Leopard (B)

### Site use by snow leopard

The top single-species occupancy model for snow leopard retained normalized difference vegetation index (NDVI) on occupancy and ruggedness on detection (Table 1).

The snow leopard niche is defined by a knife-edge response to vegetation and a strong reliance on rugged terrain for visibility (Table 2). The effect plot shows a steep threshold response (Figure 1B). Below an NDVI of 0.0 (barren rock and permanent snow), occupancy is virtually zero. As soon as NDVI becomes slightly positive, occupancy probability jumps vertically toward 1.0. This suggests that snow leopards roam across the vast landscape except areas of barren rock and permanent snow.

The ruggedness as detection driver likely reflects snow leopard’s natural behavior. They use rugged features, ridgelines, cliffs, and rocky outcrops as travel corridors, scent-marking sites (scrapes), and vantage points. Detections in rugged terrain are far more likely because the animals are funneled through these features.

The spatial occupancy map (Figure 2B) shows that 32.6% of the study area falls within the low occupancy class (0.00–0.25), 58.3% within the moderate occupancy class (0.26–0.50), and less than 9.1% within the high occupancy class (>0.50). Compared to ibex, snow leopards exhibited higher occupy in the study area.

### Multi-species occupancy

The best-supported co-occupancy model retained distance to roads (DISRD) as a key predictor of joint occupancy. Ibex occupancy was strongly influenced by distance to roads, with a significant negative effect (β = −3.69, SE = 1.83, *p* = 0.043), indicating a higher probability of occurrence closer to roads. Snow leopard occupancy exhibited a similar but non-significant trend (β = −1.12, SE = 0.88, *p* = 0.21), providing only weak evidence for greater occupancy near roads.

The species interaction term at the intercept was positive and significant (β = 4.08, SE = 2.05, *p* = 0.047), indicating that snow leopards and ibex co-occurred more frequently than expected under independence, particularly in areas closer to roads. The interaction effect of distance to roads was also positive (β = 4.47, SE = 2.33, *p* = 0.055), suggesting that although the occupancy of both species declined with increasing distance from roads, their tendency to co-occur relative to independence increased in more remote areas.

The marginal occupancy plot for ibex (Figure 3) showed a clear decline in site-use probability with increasing distance from roads, consistent with the single-species occupancy model and supporting the interpretation that ibex are primarily valley-dwelling animals. In contrast, snow leopard marginal occupancy exhibited a weak and non-linear relationship with distance to roads, indicating little evidence of a direct association with road proximity when considered independently.

**Figure 3:**
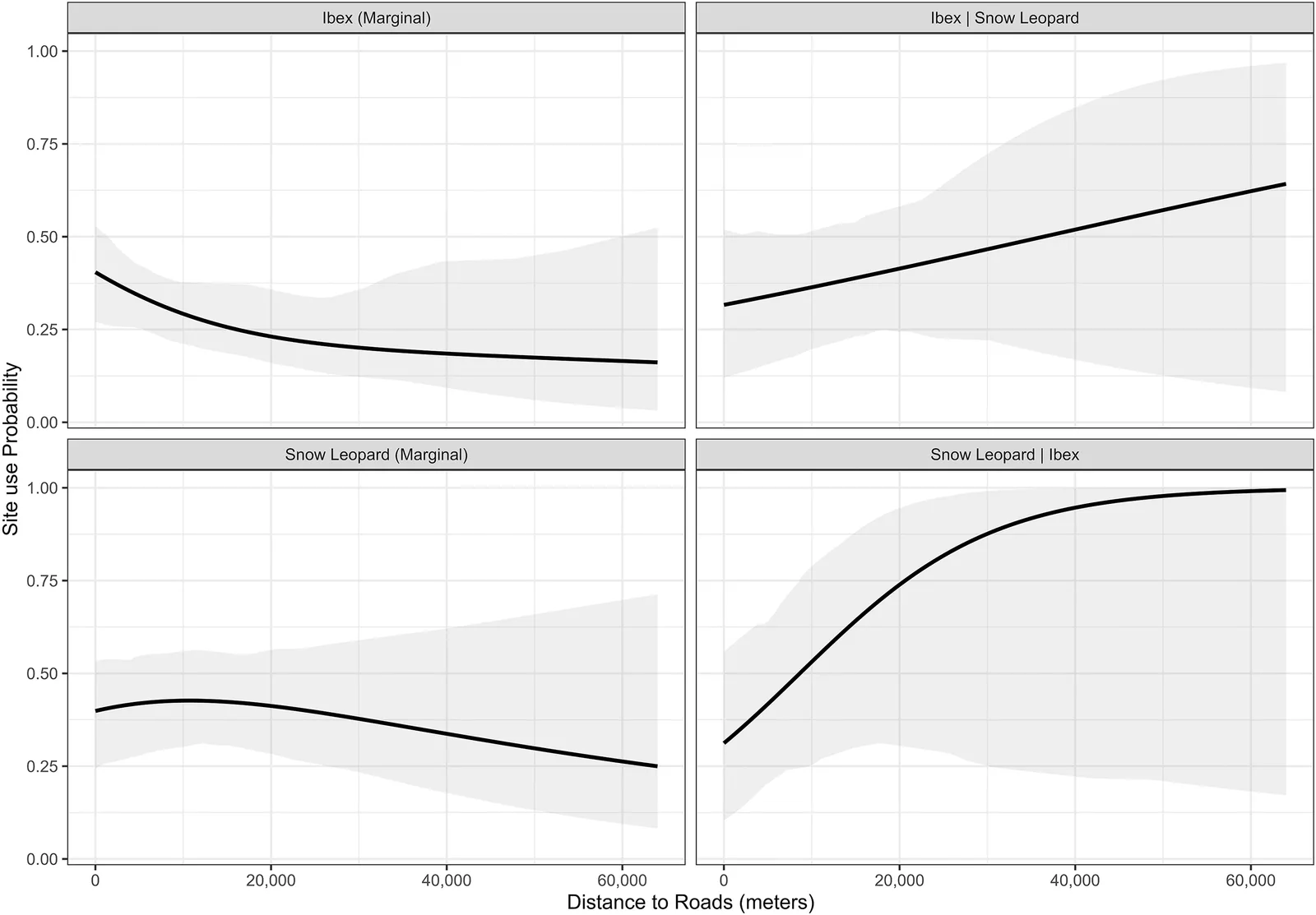
Effect plot of co-occupancy model, showing marginal and conditional occupancies of Himalayan ibex and snow leopard in response to distance to roads

Conditional occupancy patterns revealed a different response. In the presence of snow leopards, ibex site-use probability increased with distance from roads, suggesting a spatial shift in habitat use potentially associated with predator presence. Similarly, snow leopard occupancy increased markedly with distance from roads when ibex were present, reaching its highest levels at intermediate to greater distances from roads. These conditional responses suggest that spatial use by both species is influenced by their interaction, with predator occurrence closely tracking prey distribution and prey potentially adjusting habitat use in response to predation risk.

Because areas near roads also represent the most productive and vegetated portions of the landscape, they likely serve as the primary arena for predator-prey interactions. Consequently, snow leopards may be drawn into closer proximity to roads and associated infrastructure than would be expected based solely on their habitat preferences (Figure 4).

**Figure 4:**
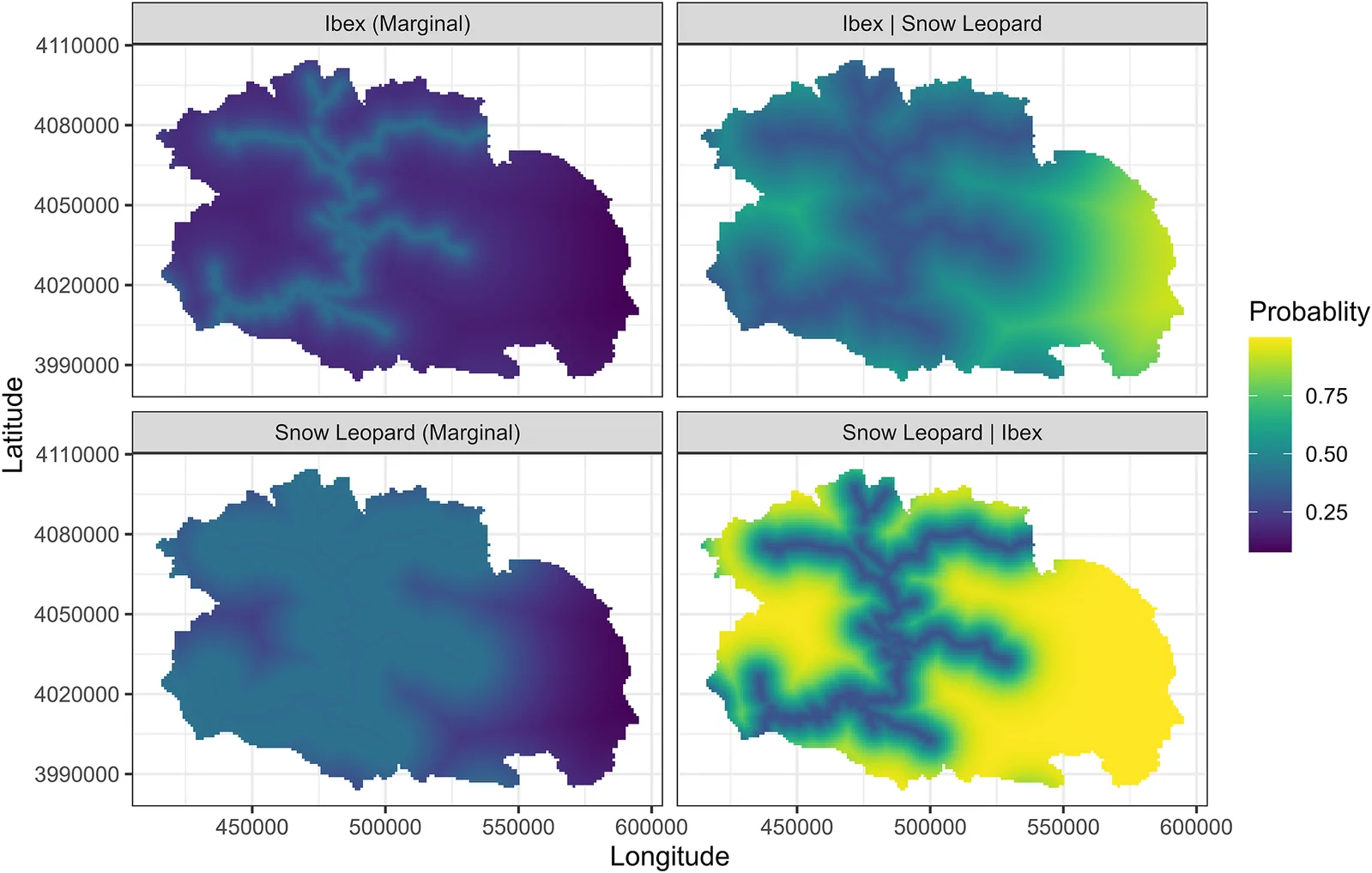
Spatial co-occupancy patterns of Himalayan ibex and snow leopard in northern Pakistan

## Discussion

Our results provide clear evidence that habitat use and co-occurrence patterns of Himalayan ibex and snow leopards in the Karakoram are shaped by both environmental gradients and interspecific interactions. The observed patterns are consistent with theoretical frameworks such as the landscape of fear, where prey species balance resource acquisition against predation risk.^20^

### Marginal occupancies

In the single-species models, ibex occupancy was positively associated with rugged terrain and higher elevations, while declining with increasing distance from roads. This finding suggests that ibex preferentially use steep, complex terrain that enhances predator detection and escape opportunities, consistent with prior studies across Central and South Asian mountain systems.^21^^,^^22^^,^^23^ The negative relationship with distance to roads may reflect indirect ecological effects rather than attraction to infrastructure. In high-altitude environments such as the Karakoram, roads often follow valley bottoms where forage availability and accessibility are higher, potentially making these areas attractive to herbivores despite associated risks.^24^

Snow leopard detection and occupancy patterns further emphasize the importance of terrain structure. Detection probability increased significantly in rugged landscapes, supporting the well-established view of snow leopards as ambush predators that rely on broken terrain to stalk prey effectively.^25^^,^^26^ Although NDVI showed a positive relationship with snow leopard occupancy, the species exhibited limited association with highly productive vegetation areas. This likely reflects the spatial configuration of vegetation in the study region, where productive patches are restricted and may not coincide with optimal hunting.^27^^,^^28^

### Conditional occupancies

The multi-species occupancy models revealed important insights into predator-prey spatial dynamics. The significant positive interaction term indicates that co-occurrence between ibex and snow leopards is higher than expected under independence, suggesting biologically meaningful spatial overlap. However, this overlap is not static; rather, it is mediated by environmental factors, particularly distance to roads. While ibex showed reduced occupancy with increasing distance from roads, the strength of co-occurrence increased in more remote areas. This pattern suggests that predator-prey interactions intensify away from human-modified landscapes, where ecological processes operate with fewer anthropogenic constraints.^29^^,^^30^^,^^31^^,^^32^

Notably, habitat response curves indicate a shift in space use under co-occurrence conditions. Ibex exhibited increased site-use probability farther from roads when snow leopards were present, suggesting a behavioral adjustment to reduce predation risk. Such spatial displacement is a common anti-predator strategy and has been widely documented in ungulates exposed to large carnivores.^33^^,^^34^^,^^35^ Conversely, snow leopards showed a strong increase in site-use probability with increasing distance from roads in the presence of ibex, indicating that predator spatial distribution is closely linked to prey movements. This supports the notion that large carnivores exhibit adaptive habitat selection in response to prey availability, a pattern observed in multiple carnivore ungulate systems.^16^^,^^36^ Taken together, these findings demonstrate that co-occurrence between ibex and snow leopards is driven by habitat-mediated processes and behavioral feedback rather than passive overlap.

Ibex select areas that balance forage availability and safety, while snow leopards track prey distributions within structurally complex environments that facilitate hunting success. This dynamic interaction highlights the importance of integrating species interactions into habitat models, as single-species approaches may overlook critical ecological mechanisms.^37^^,^^38^

### Implications for conservation planning

From a conservation perspective, the results underscore the ecological importance of heterogeneous mountain landscapes that provide both refuge for prey and hunting grounds for predators. The apparent concentration of predator-prey interactions in more remote areas suggests that minimizing human disturbance in these zones could be critical for maintaining natural ecological processes. Furthermore, the association of both species with areas near roads in single-species contexts highlights potential conservation challenges, as expanding infrastructure in high-mountain regions may alter habitat use and disrupt predator-prey dynamics.

Overall, this study contributes to a growing body of evidence demonstrating that predator and prey distributions in alpine ecosystems are tightly coupled through both environmental constraints and behavioral adaptations. Multi-species occupancy approaches provide a powerful framework for disentangling these relationships and should be further applied to guide conservation planning in fragile mountain ecosystems.

### Limitations of the study

While interpreting the findings of this study, several important limitations should be considered. First, the analysis is based on a “snapshot” occupancy framework, which effectively captures spatial patterns of site use but does not account for temporal avoidance or dynamic interactions among species; consequently, short-term behavioral responses occurring at finer temporal scales may not be fully represented.^18^^,^^38^ Second, although NDVI was employed as a proxy for vegetation productivity, its reliability may be influenced by the presence of snow cover, which can alter spectral reflectance and potentially bias estimates of habitat quality in alpine or seasonally snow-covered landscapes.^39^ Third, the analysis assumes population closure during the sampling period implying no immigration, emigration, births, or deaths, yet violations of this assumption can affect both detection probabilities and occupancy estimates.^38^^,^^40^ Finally, uncertainty in model selection and parameter estimation remains an inherent challenge, particularly when competing models receive comparable support or when sample sizes are limited, potentially influencing the robustness and generality of the results.^41^ Collectively, acknowledging these constraints provides essential context for interpreting the study’s findings and underscores the need for cautious inference.

### Conclusion

This study demonstrates that Himalayan ibex habitat selection is strongly influenced by perceived predation risk, as evidenced by their preference for rugged, high-elevation terrain near rivers but avoidance of greener areas. Snow leopard occupancy also varied with habitat features but appeared to adjust to prey distribution under certain conditions. These findings highlight how prey behavior shapes predator movement in heterogeneous landscapes and provide further evidence for the landscape of fear.

Multi-species occupancy models proved effective for exploring spatial dynamics in predator-prey systems and offer a powerful framework for future ecological and conservation studies in mountainous regions.

## Resource availability

### Lead contact

Further information and requests for resources should be directed to and will be fulfilled by the lead contact, Muhammad Ali Nawaz (nawazma@gmail.com).

### Materials availability

This study did not generate new unique reagents.

### Data and code availability

• All data reported in this paper and R code are available at the following repository: https://data.mendeley.com/preview/cgcp7pkjpp?a=301694b7-747b-4f17-ae96-69f86854a543.
• Any additional information required to reanalyze the data reported in this article is available from the lead contact upon request.

## Acknowledgments

We acknowledge the support provided by the staff of Parks & Wildlife Department, Gilgit-Baltistan and local communities during the field surveys. Financial support for the data collection came from Snow Leopard Trust; Higher Education Commission-Pakistan bore the expenses to visit the 10.13039/100007919University of Massachusetts to conduct analysis of this paper under the “International Research Support Initiative Program (IRSIP).” We also appreciate the assistance provided by the SLF staff and students of 10.13039/501100004253Quaid-i-Azam University during our fieldwork, particularly Mohammad Younas, Shoaib Hameed, and Muhammad Kabir.

## Author contributions

H.A., C.S., M.A.N., conceptualization, investigation, data curation, writing – original draft, formal analysis, methodology, software, visualization, and validation; J.U.D., data curation and writing – review and editing. M.A.N., supervision, project administration, and funding acquisition.

## Declaration of interests

The authors declare that they have no known competing financial interests or personal relationships that could have appeared to influence the work reported in this paper.

## STAR★Methods

### Key resources table

**Table:** 

REAGENT or RESOURCE	SOURCE	IDENTIFIER
**Deposited data**		

DEM	EarthExplorer, USGS	https://earthexplorer.usgs.gov/
NDVI	GloVis, USGS	https://glovis.usgs.gov/
TRI	Riley, DeGloria, Elliot^42^ (Using ArcHydro Tools 3.2.2).	https://arc.lib.montana.edu/ojs/index.php/IJS/article/view/2914
Rivers	Digitized	https://googleearth.com
Roads	Digitized	https://googleearth.com

**Software and algorithms**		

ArcGIS Pro	ESRI	https://www.esri.com/
R	R Core Team	https://www.r-project.org/; RRID: SCR_001905
unmarked package (v 1.5.2)	unmarked: Models for Data from Unmarked Animals	https://CRAN.Rproject.org/package=unmarked
ArcHydro Tools 3.2.2	ESRI	https://www.esri.com/en-us/industries/water-resources/arc-hydro

### Method details

#### Study area

This study was conducted across multiple valleys in the Hunza and Nagar districts of northern Pakistan, including Khunjerab, Misgar, Shimshal, Khudabad, Khyber, Hussaini, Gulmit, Ghulkin, Ahmedabad, Hassanabad, Nagar Khas, Hopper, and Hisper. These valleys lie along the Pakistan–China border between 74°E to 76°E longitude and 36°N to 37°N latitude, covering an area of approximately 14,826 km^2^. The region is characterized by highly rugged topography with altitudinal variation from 1,695 to 7,889 m above sea level (Figure 5).

**Figure:**
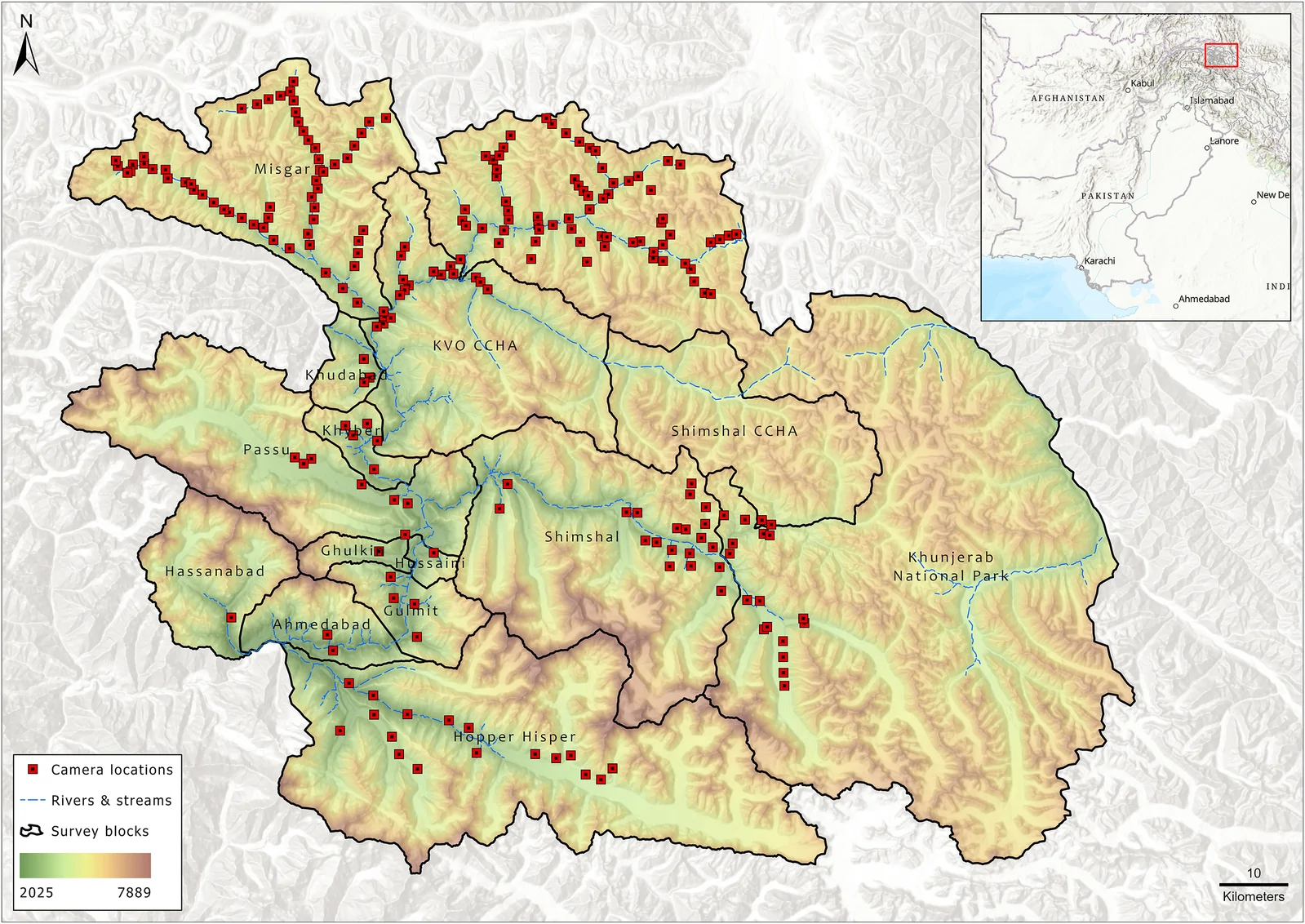
Study area showing locations of camera sites in northern Pakistan

The climate is cold and arid, with average monthly temperatures dropping to −1°C during winter (November–February) and reaching up to 23.3°C in summer (June–August).The lowest winter temperature reaches −18°C, while the highest summer temperature rises to 30°C. Annual precipitation averages 149.7 mm, mostly falling as snow in winter, with occasional rainfall in March and April.^43^ Due to the barrier effect of the Himalayas and Hindu Kush ranges, the Karakoram landscape receives minimal influence from the monsoon system,^44^ supporting a dry alpine steppe ecosystem. These valleys provide habitat for diverse flora (e.g., *Artemisia* spp.*, Primula macrophylla, Saxifraga sibirica*) and fauna, including snow leopards (*Panthera uncia*), Himalayan ibex (*Capra sibirica*), brown bear (*Ursus arctos isabellinus*), gray wolf (*Canis lupus*), red fox (*Vulpes vulpes*), and blue sheep (*Pseudois nayaur*).^45^

#### Camera trapping

We deployed 224 infrared/motion-triggered cameras (Reconyx HC 500 and HC 900; Reconyx, Holmen, USA), using 5 × 5 km grids as sampling units, cameras were placed in each accessible grid. A minimum spatial distance of 1 km was maintained between cameras to maximize spatial coverage given terrain constraints. Although this spacing is smaller than the typical home range of snow leopard and ibex, as occupancy models estimate site-level probability of use rather than individual presence, and therefore do not require strict independence among sites. Furthermore, this spacing was consistent with the previous camera-trap occupancy studies in similar systems and is appropriate for inference at the scale of sampling unit.^11^^,^^36^ The cameras were mounted on specially designed 40–60 cm iron stands.^46^ The presence of any indirect animal signs, e.g., scats, scrapes, pugmarks, chin rubs, and pellets,^47^ and habitat features like ridges, cliff bases, and draws were considered important locations for camera installation.^46^ To increase the detectability of target species, lure stations were established one meter away from each camera pole in view of the camera by leveling the ground (useing foot). Plaster of Paris tablets soaked in fish oil were placed on the leveled ground while boulder or vegetation was used to apply caster or skunk. These lures enhance the detectability of carniovres while not attracting herbivores.^46^^,^^48^ Using camera trapping survey formats, information like camera locations (Latitude and Longititude), type of lure, camera site potential, and signs of animals in a 20-meter buffer, were recorded.

The cameras were left operational for 15–80 days across four camera-trap surveys. The surveys were conducted as follows: (1) from 21 September – 9 November 2011, during which each cameras was opertioanl for 15 nights and was then moved to the next site for another 15 nights; (2) from 11 May – 13 June 2013, with a samping duration of 30 days; (3) from 3 March – 7 May 2016, with cameras active for 65 days; and (4) from 11 April – 1 July 2018, lasting 80 days. Trap histories of Himalayan ibex and snow leopards were constructed by assigning a value of ‘0’ for absence and ‘1’ for the presence at each camera site. To reduce zero inflation in the dataset, detection/non-detection data collected over the 80-day survey period were aggregated into 3-day periods for each camera station, resulting in 27 sampling occasions.^36^

#### Covariates

Ibex and snow leopard inhabit sites based on certain habitat features. Therefore, we included both natural (elevation, slope, aspect, distance to rivers, distance to glaciers, and normalized difference vegetation index (NDVI) and manmade variables (distance to roads). Shuttle Radar Topography Mission (SRTM) data was downloaded from (https://earthexplorer.usgs.gov/). The Euclidean distances of rivers, glaciers, and roads (vehicular roads) and NDVI were calculated using Band 04 and Band 05 of Landsat 8 imagenery for peak growing season (April - August), (downloaded from (https://glovis.usgs.gov/), while slope and aspect were dervied form STRM using the specific tools for each operation in the spatial analyst tool of ArcGIS Pro 3.2.2 (ESRI Inc, Redlands, USA), finally the Terrain Ruggedness Index (TRI) was derived from the slope and aspect.^42^ All the enviromental layers were avaialbe at 30 m resolution were upscaled to 1000m to align the spatial resolution of predictors with the scale of camera-trap occupancy sites and to reduce spatial mismatch between fine-scale environmental data.

We extracted aforementioned co-variates using a circular buffer of 1,000 m around each camera station,^49^ these circular buffer encompaes an area of 3.14 km^2^. Telemetric study on ibex reavled that ibex concenterated their majority of activities usually in 4.76 km^2^.^50^ The average value of each variable was extracted using the zonal statistics tool in ArcGIS Pro 3.2.2. The values were standardized at the zero mean and standard deviation of 1^51^ using the standardized function of the psycho package in R.^52^ We did not use any two variables that were correlated >0.70 using the Pearson correlation test to avoid multi-collinearity among variables (Figure S1).^53^

#### Occupancy modeling

We first ran a single-season, single-species occupancy model developed by.^18^ We hypothesized the need for a distinct set of habitat covariates as the species under consideration have different evolutionary lineages and dietary requirements. Therefore, we first checked the effect of different habitat covariates on the detection probability of ibex and snow leopards. The covariates from the best detection model were kept constant when running models for estimating occupancy^54^ using the occu function of the unmarked package in R.^55^ We then selected habitat covariates to use in multi-species models from the best detection and occupancy model using the Akaike Information Criteria (AIC).^41^ We used a multi-species occupancy model^19^ to assess whether Himalayan ibex and snow leopard co-occur more or less than expected by chance given their individual habitat preferences.^56^ Co-occurrence models were conducted using the occuMulti function of the unmarked package in.^52^ Models are powerful as they can estimate the impact of Himalayan ibex and snow leopard on each other’s occupancy against the impact of different covariates. We kept the detection and occupancy state covariates taken from single-species models fixed, we then modeled the conditional occupancy probabilities (e.g., probability of ibex occupancy given snow leopard presence). We adopted the model selection approach proposed and advocated for by^57^ which has been shown to recover the appropriate top model. Moreover, a recent paper^58^ has demonstrated that when prediction is the aim, the multispecies co-occurrence model is appropriate for predicting ecological states (but admittedly less reliable for inference). We then ranked multi-species models using AIC to find the best model.^41^^,^^57^^,^^59^

### Quantification and statistical analysis

Single-species and multi-species occupancy models were used to quantify site-use patterns of the Himalayan ibex and snow leopard. The best-performing single-species and multi-species occupancy models were ranked using the Akaike Information Criterion (AIC).^41^
