# Supplementary material for: Greenery or fear: Site use of Himalayan ibex in a predator-dominant landscape
Source: iScience. 2026 Oct 22;29(11):117885. doi: 10.1016/j.isci.2026.117885 (PMC13718294; doi:10.1016/j.isci.2026.117885)
Supplement: Supplementary file 1 [file mmc1.pdf]

iScience, Volume *29*

Supplemental information

Greenery or fear: Site use of Himalayan ibex in a predator-dominant landscape

Hussain Ali, Chris Sutherland, Jaffar Ud Din, and Muhammad Ali Nawaz

Supplemental information

Greenery or fear: site use of Himalayan ibex in a predator-dominant landscape

Hussain Ali, Chris Sutherland, Jaffar Ud Din, Muhammad Ali Nawaz

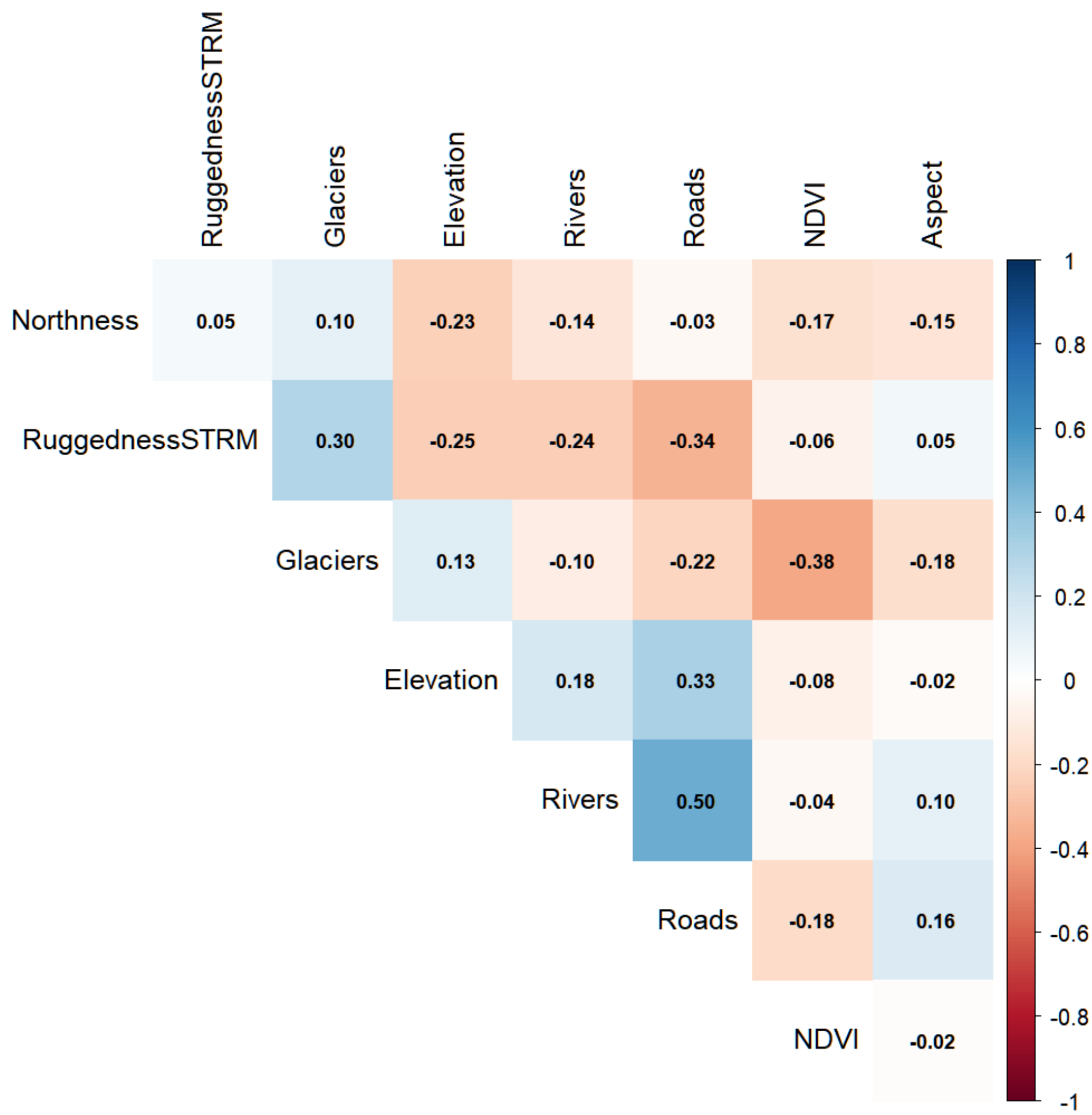

Figure S1. Correlation matrix of the variables used in the occupancy models.
